# Response to comments on: Comparison of suprapatellar versus infrapatellar approaches of intramedullary nailing for distal tibia fractures

**DOI:** 10.1186/s13018-021-02263-2

**Published:** 2021-02-05

**Authors:** Yao Lu, Gen Wang, Bin Hu, Cheng Ren, Liang Sun, Zhimeng Wang, Changjun He, Hanzhong Xue, Zhong Li, Kun Zhang, Teng Ma, Qian Wang

**Affiliations:** 1 Department of Orthopaedic Surgery, HongHui Hospital, Xi’an Jiaotong University, 555 Youyi East Road, Xi’an, 710054 Shaan’xi Province China; 2 Bioinspired Engineering and Biomechanics Center (BEBC), School of Life Science and Technology, Xi’an Jiaotong University, Xi’an, 710049 China; 3 Orthopaedics Institute of Chinese PLA, 80th Hospital, 3770 Beigongxijie, Weifang, Shandong Province China; 4 Department of Hematology, Xi’an Gao Xin Hospital, Xi’an, 710054 Shaan’xi Province China; 5 Yan’an University, Yan’an, 710000 Shaanxi China; 6 Department of Orthopaedic Surgery, HongHui Hospital, Xi’an Jiaotong University, 555 Youyi East Road, Xi’an, 710054 Shaan’xi Province China; 7 Bioinspired Engineering and Biomechanics Center (BEBC), School of Life Science and Technology, Xi’an Jiaotong University, Xi’an, 710049 China; 8 Department of Orthopaedic Surgery, HongHui Hospital, Xi’an Jiaotong University, 555 Youyi East Road, Xi’an, 710054 Shaan’xi Province China

Thanks very much for your interest in the article “Comparison of suprapatellar versus infrapatellar approaches of intramedullary nailing for distal tibia fractures” [1]. We agree with Dr. Lu’s opinion that the description of the ideal starting point and Fig. 2B was not matched in our manuscript. In order to strength our statement, we provided a more rigorous Figure to match our opinion that the ideal starting point for tibial nailing of the SP approach is in the intersection of tibial midline and tibial plateau articular surface. As demonstrated by Bible et al. [2], twin peaks anteroposterior position (AP) images is safe for a starting point according to tibia-based referencing for proximal tibia radiographs. Too lateral a start can cause varus, in the tibia fractures, whereas too medial a starting point can create valgus [2]. Therefore, we recommend the ideal enter point as being “in the intersection of tibial midline and tibial plateau articular surface” at AP image and at the anterior edge of the tibial plateau on the lateral view. The ideal starting point in Fig. 2B we provided is slightly lower. We now correct Fig. 2B to provide a more accurate lateral starting point.

Replaced Figure 2B Starting point under fluoroscopic guidance

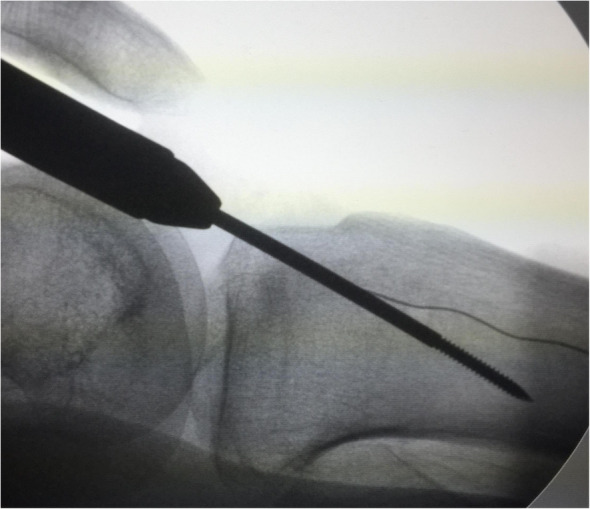



## Data Availability

Not applicable.
